# Use of fluorescence imaging to optimize location of tissue sampling in hard-to-heal wounds

**DOI:** 10.3389/fcimb.2022.1070311

**Published:** 2023-01-12

**Authors:** Thomas E. Serena, Robert J. Snyder, Philip G. Bowler

**Affiliations:** ^1^ SerenaGroup Research Foundation, Cambridge, MA, United States; ^2^ Foot and Ankle Institute, Barry University, Miami, FL, United States; ^3^ Phil Bowler Consulting, Warrington, England, United Kingdom

**Keywords:** wound infection, biopsy, sampling technique, microbiological analysis, antibiotic stewardship, diagnostic stewardship, fluorescence imaging, point-of-care diagnostic

## Abstract

**Introduction:**

Wound microflora in hard-to-heal wounds is invariably complex and diverse. Determining the interfering organisms(s) is therefore challenging. Tissue sampling, particularly in large wounds, is subjective and, when performed, might involve swabbing or biopsy of several locations. Fluorescence (FL) imaging of bacterial loads is a rapid, non-invasive method to objectively locate microbial hotspots (loads >10^4^ CFU/gr). When sampling is deemed clinically necessary, imaging may indicate an optimal site for tissue biopsy. This study aimed to investigate the microbiology of wound tissue incisional biopsies taken from sites identified by FL imaging compared with sites selected by clinical judgment.

**Methods:**

A *post hoc* analysis of the 350-patient FLAAG wound trial was conducted; 78 wounds were included in the present study. All 78 wounds were biopsied at two sites: one at the center of the wound per standard of care (SoC) and one site guided by FL-imaging findings, allowing for comparison of total bacterial load (TBL) and species present.

**Results:**

The comparison between the two biopsy sites revealed that clinical uncertainty was higher as wound surface area increased. The sensitivity of a FL-informed biopsy was 98.7% for accurately finding *any* bacterial loads >10^4^ CFU/g, compared to 87.2% for SoC (p=0.0059; McNemar test). Regarding species detected, FL-informed biopsies detected an average of 3 bacterial species per biopsy versus 2.2 species with SoC (p < 0.001; t-test). Microbial hotspots with a higher number of pathogens also included the CDC’s pathogens of interest.

**Conclusions & perspective:**

FL imaging provides a more accurate and relevant microbiological profile that guides optimal wound sampling compared to clinical judgment. This is particularly interesting in large, complex wounds, as evidenced in the wounds studied in this *post hoc* analysis. In addition, fluorescence imaging enables earlier bacterial detection and intervention, guiding early and appropriate wound hygiene and potentially reducing the need for antibiotic use. When indicated, this diagnostic partnership with antibiotic stewardship initiatives is key to ameliorating the continuing threat of antibiotic resistance.

## 1 Introduction

Hard-to-heal wounds afflict millions of people worldwide and are a significant burden to patients, caregivers, and healthcare institutions (Sen et al., 2009; Sen, 2021). Challenges in their treatment stem from the diverse co-morbidities of the typical chronic wound population that is largely elderly and immunocompromised (Lipsky et al., 2016). Patients with these characteristics usually fail to mount adequate immune responses, resulting in a lack of identifiable clinical signs and symptoms of infection and inflammation (CSS). These CSS would alert the clinicians, and ideally flag the locations of the wound burdened and at risk, harboring biofilm, high bacterial loads, and/or infection, but that is seldom the case (Cant and Cole, 2010). When they do present, CSS of infection in chronic wounds are often atypical, such as wound deterioration or new onset of pain in an otherwise painless foot in the case of DFUs (Gardner et al., 2001; Gardner et al., 2007). These are unspecific and do little to help precisely localize the bacterial hotspot(s). These challenges impede clinicians’ decision-making capabilities regarding procedures as fundamental and routine as when and where to debride, and as advanced and specialized as selection of the ideal sites for grafting and sampling.

Sampling for microbiology in complex, chronic wounds have specific indications and can be done through various sample collection methods such as swabs (surface or Levine technique), biopsies, or by the analysis of wound fluids and/or the tissues removed during debridement. Ideally, sampling should be reserved for certain indications (e.g., prior to grafting, prior to cellular and tissue-based product (CTP) placement, or to determine antibiotic sensitivities when there is reason to prescribe); sampling should not be used indiscriminately. When medically indicated, determining the ideal location(s) to sample a wound maximizes the benefit of this procedure. Misuse of resources, including failed grafts and CTPs (Unal et al., 2005) as well as the excessive and inappropriate use of antibiotics (Serena et al., 2022) and other antimicrobials, negatively impacts outcomes and increases morbidity. Antibiotic use in wound care is higher than most other medical fields (Lipsky et al., 2016). This defies evidence advocating for preferentially local treatments such as wound hygiene and debridement (Sen et al., 2009; Sen, 2021) and is predicted to contribute to the global escalation in antibiotic resistance (Bowler et al., 2020).

That these limitations have yet to be overcome by the chronic wound standard of care suggests that an objective and technologically supported method may be needed. The use of technological aids to guide and bolster diagnostic efforts and aid antibiotic stewardship initiatives has evolved into the concept of *diagnostic stewardship* (WHO, 2016; Patel and Fang, 2018; Sullivan, 2021). Across medical fields the diagnostic stewardship initiative has been introduced as a key element for the appropriate use of laboratory testing – both whether to perform the testing and, if so, in what manner - to guide patient diagnosis, management, and treatment selection. This initiative has organically evolved into a symbiotic partner to antibiotic stewardship.

Adopting technology, like FL-imaging portable devices, to enhance sampling location selection and therefore the *diagnostic accuracy* of wound microbiological analysis is in line with diagnostic stewardship principles. Point-of-care FL-imaging alerts to the location of high bacterial loads (Serena et al., 2008; Rennie et al., 2017; Raizman, 2019; Raizman et al., 2019; Le L et al., 2021). The device detects most bacterial pathogens and has a PPV ranging between 93-100%, depending on the pathogen(s) present (Rennie et al., 2017; Jones et al., 2020; Le L et al., 2021; Raizman et al., 2021). Red fluorescence indicates the presence of endogenously produced porphyrins from most bacteria (including Gram negatives/positives, anaerobes and aerobes) at loads >10^4^ CFU/g (Jones et al., 2020), while cyan fluorescence indicates *Pseudomonas aeruginosa* (Raizman et al., 2021). The present *post hoc* analysis that focuses on the biopsy samples obtained in the 14-site, 350-wound prospective Fluorescence Imaging Assessment and Guidance (FLAAG) clinical trial (Le L et al., 2021). This trial was designed to measure the diagnostic accuracy and utility of this technology in the management of chronic wounds. The primary outcomes of the FLAAG trial demonstrated that the imaging procedure provided accurate, real-time mapping of areas of high bacterial loads greater than 10^4^ CFU/g in and around wounds, with a sensitivity 4 to 11-fold greater than CSS alone (standard of care diagnosis) (Rennie et al., 2017; Le L et al., 2021; Sandy-Hodgetts K et al., 2021). In this study, we are leveraging the information collected during the FLAAG trial from a subset of 78 wounds from which two biopsies were taken. One biopsy was taken as per standard of care (SoC) guidance (i.e., center of the wound), and the second performed on a positive area of FL. We compare the total bacterial loads (TBL) and the number and type of pathogens captured at each biopsy location (SoC versus FL). Additionally, we compare the wound and patient characteristics between this two-biopsy cohort with the remaining FLAAG trial population to determine potential factors that could predict cases in which SoC sampling location is more likely to be inaccurate.

## 2 Methods

### 2.1 Patient population and design

**Figure 1:**
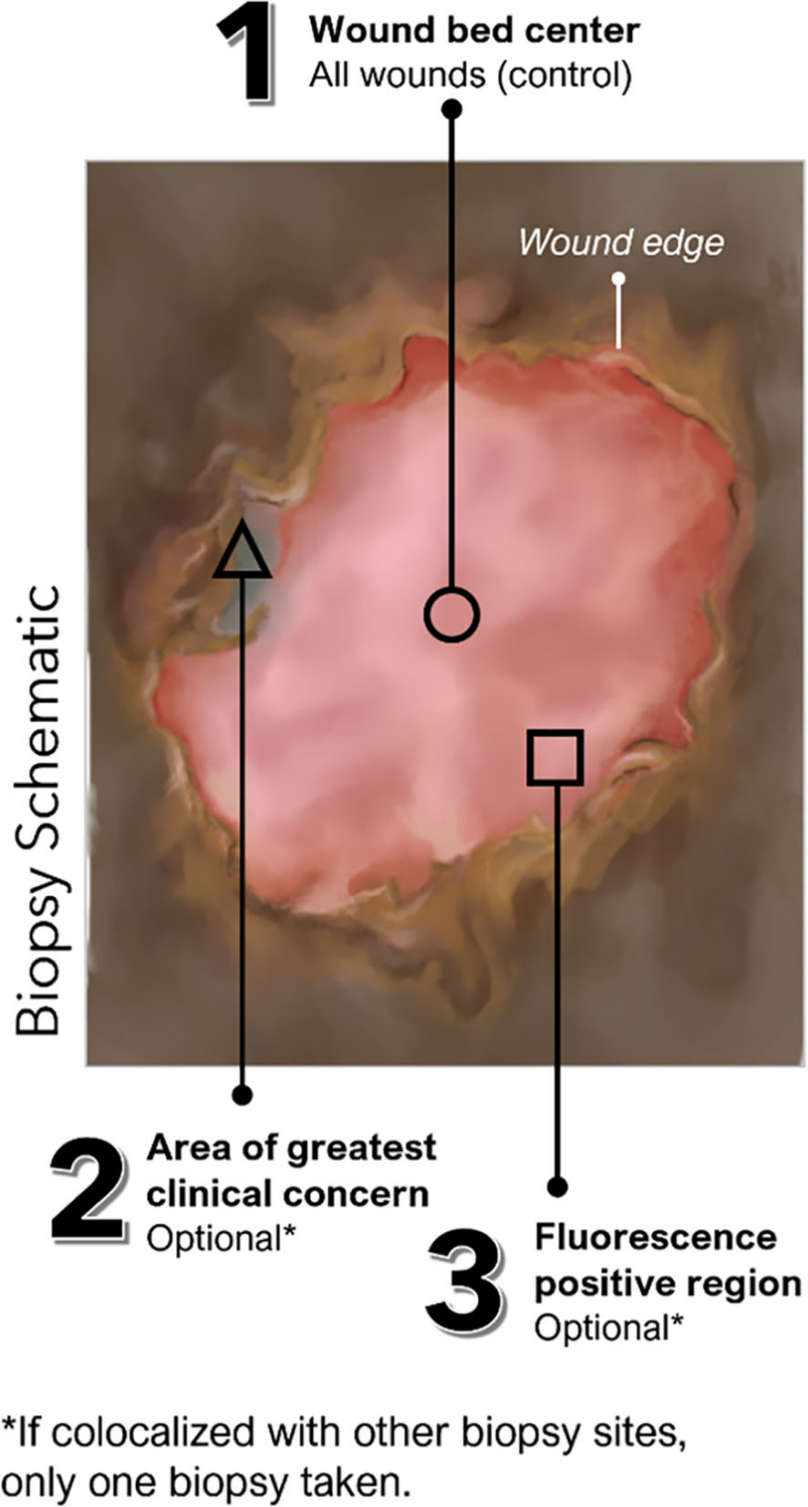
Possible locations of tissue biopsies collected in FLAAG trial. (Schematic*)*: Up to 3 tissue biopsies could have been collected per wound including (1): Standard of Care, at the center of wound (mandatory per trial protocol) (2); an area of interest with suspicion of bacterial presence as indicated by CSS (CSS+) (of note, there were no tissue samples obtained from locations purely based on CSS); and/or (3) an area of the wound and surrounding region positive for red or cyan fluorescence (FL+).

This prospective, single-blind, multi-center, cross-sectional clinical trial included 350 wounds with unknown infection status. Exclusion criteria for this trial (clinicaltrials.gov#NCT03540004) included: (a) treatment with an investigational drug within 1 month before study enrollment, (b) recent (<30 days) biopsy or curettage of target wound, (c) wounds that cannot be completely imaged by study device due to anatomic location, (d) inability to consent, or (e) any contra-indication to routine wound care and/or monitoring. In the FLAAG trial, a single biopsy was captured for 272 wounds, while 78 had two biopsies performed. The reason for this double-biopsy requirement in the 78-wound subset was that while one of the sample sites was determined by SoC (center of the wound), FL-imaging findings and/or clinical signs and symptoms (CSS) of infection outside of the center of the wound (SoC) could grant the collection from that additional site. This was done at the clinician’s discretion. In this trial all additional sites corresponded to a FL-positive site and not to an area deemed CSS (+). A detailed description of sample locations is provided in **Figure 1**.

### 2.2 Clinical assessment and fluorescence imaging procedure

All clinicians involved were highly experienced in the management of chronic, hard-to-heal wounds, and practiced at one of 14 different specialized outpatient wound clinics across the USA. All those involved in sample site selection based on FL-imaging findings had to undergo in-depth training on the use of the device and image interpretation and trial procedures before commencing the study.

The sequence of image acquisition, interpretation, and decision making was as follows: 1) clinical evaluation, measurement of wound, and capture of standard (non-fluorescent) image (ST-image), followed by selection of biopsy site(s) based on standard of care; 2) preparation for FL-imaging (e.g. darkened environment, device positioning at an appropriate distance for imaging validated by range finder indicator), image capture, image interpretation relative to the ST-image, and selection of biopsy site(s) based on FL-imaging findings. This sequence was followed in the same, strict order as per the trial’s protocol to prevent biased sample site selection. Wounds where the SoC sample site (center of the wound) overlapped with a FL imaging positive site were noted but were assigned to the single-biopsy cohort. Both ST and FL-images were obtained using the MolecuLight *i:*X (MolecuLight, Toronto, Canada).

### 2.3 Microbiological analysis in the FLAAG clinical trial protocol

Thorough cleansing of the wound to remove debris and surface contamination was performed with saline and gauze on each wound prior to biopsy. Otherwise, the sample site was prepared as per each institution’s protocol (e.g., use of lidocaine). Punch biopsies (6 mm diameter, trimmed to 2 mm depth) were selected as the gold-standard to uniformly obtain samples for quantitative analysis of total bacterial load to meet the trial’s primary endpoints (diagnostic accuracy metrics (Le L et al., 2021).

Detailed description of the microbiological methods used were reported in a previous publication by Serena et al. (Serena TE et al., 2021). In brief, each biopsy was transported in Remel ACT II media to a CLIA-certified central laboratory (Eurofins Central Laboratory, Lancaster, PA); transport time ranged between 24-48 hours. Use of non-nutritive medium provided a protective environment that preserved the specimens while preventing bacterial growth during transportation. The laboratory used gold standard, aseptic techniques for analysis of load and species. Quantitative culture was performed as previously described, with every effort made to provide optimal conditions for bacteria that are challenging to culture (e.g., anaerobes). Diluted biopsy samples were cultured on various agars to support aerobic and anaerobic growth (Le L et al., 2021; Serena TE et al., 2021). Matrix assisted laser desorption ionization-time of flight (MALDI-TOF) mass spectrometry (Bruker Daltonics) was used to identify bacterial species. Microbiologists were blinded to the results of the CSS assessment and FL-imaging.

### 2.4 Statistical analysis

Wound and patient characteristics were compared for statistically significant differences through two tailed t-tests. The effectiveness of FL-informed biopsies versus SoC biopsies were compared by evaluating how: 1) Fluorescence imaging performs in the *detection of total bacterial load* at levels ≥10^4^ CFU/g; 2) Fluorescence imaging performs in the *detection of specific pathogens* at levels ≥10^4^ CFU/g.

FL positive and negative samples from the 78 specimens were analyzed for sensitivity; specificity was not calculated as these were all microbiologically positive samples. Because each wound was evaluated by two methods, SoC and FL-imaging, a correlated binomial distribution for sensitivity method was used. 95% Clopper Pearson 2-sided confidence limits were calculated for these proportions. The McNemar test was used to determine statistical significance. A second analysis determined if the number of pathogens was greater in the FL-informed biopsy sample than in the SoC sample through a t-paired test. Two endpoints were measured (Sen, 2021): the total number of bacterial species, and (Sen et al., 2009) the total number of species considered antibiotic resistant threats by the CDC (2019a) as a marker of relevance for a more expansive, precise sample.

## 3 Results

### 3.1 Description of the study’s subset

A detailed description of the study population is provided in **Table 1**, as well as a comparison between the double-biopsy cohort (n=78) and the single-biopsy cohort (n=272). There was no significant difference in age (*p=*0.48) or sex distribution (*p=*0.11), however, the history of recent systemic antibiotic prescription prior to their enrolment in the FLAAG clinical trial was significantly different between the cohorts (*p=*0.007), being greater in the single-biopsy group (14% vs 29%). The rationale for and/or clinical significance of the latter finding is unclear and beyond the scope of the present *post-hoc* analysis. **Table 1** also provides an overview of the wounds included in each group; there was a homogeneous distribution in etiologies of the wounds, their duration, and their location.

**Table 1:** Comparison of patient and wound characteristics between double-biopsy and single-biopsy cohorts.

	Double-biopsy cohort	Single-biopsy cohort	
**Patient subset overview**			
**Age**	59.32 (SD 12.33) [34-92]	60.44 (SD 12.48) [28-96]	*p*=0.48
**Sex (Female)**	22/78 (28.21%)	103/272 (37.87%)	*p*=0.117
**Systemic antibiotic prescribed**	11/78 (14.1%)	79/272 (29%)	p=0.007
Wound subsets overview			
**Wound Type**			*p=*0.48
DFU	33/78 (42.31%)	105/272 (38.60%)	
PU	6/78 (7.69%)	16/272 (5.88%)	
SSI	10/78 (12.82%)	50/272 (18.38%)	
VLU	22/78 (28.21%)	84/272 (30.88%)	
Other*	7/78 (8.97%)	17/272 (6.25%)	
**Wound Duration**			*p=* 0.2615
<3 Months	22/78 (28.21%)	84/272 (30.88%)	
3-12 Months	23/78 (29.49%)	95/272 (34.93%)	
> 12 Months	33/78 (42.31%)	93/272 (34.19%)	
**Wound Location**			*p=*0.2196
Trunk	5/78 (6.41%)	15/272 (5.51%)	
Limb	70/78 (89.74%)	220/272 (80.88%)	
Other**	3/78 (3.85%)	37/272 (13.60%)	

*Other aetiologies included traumatic, vasculitic and burns.

**Other locations included.

**Figure 2:**
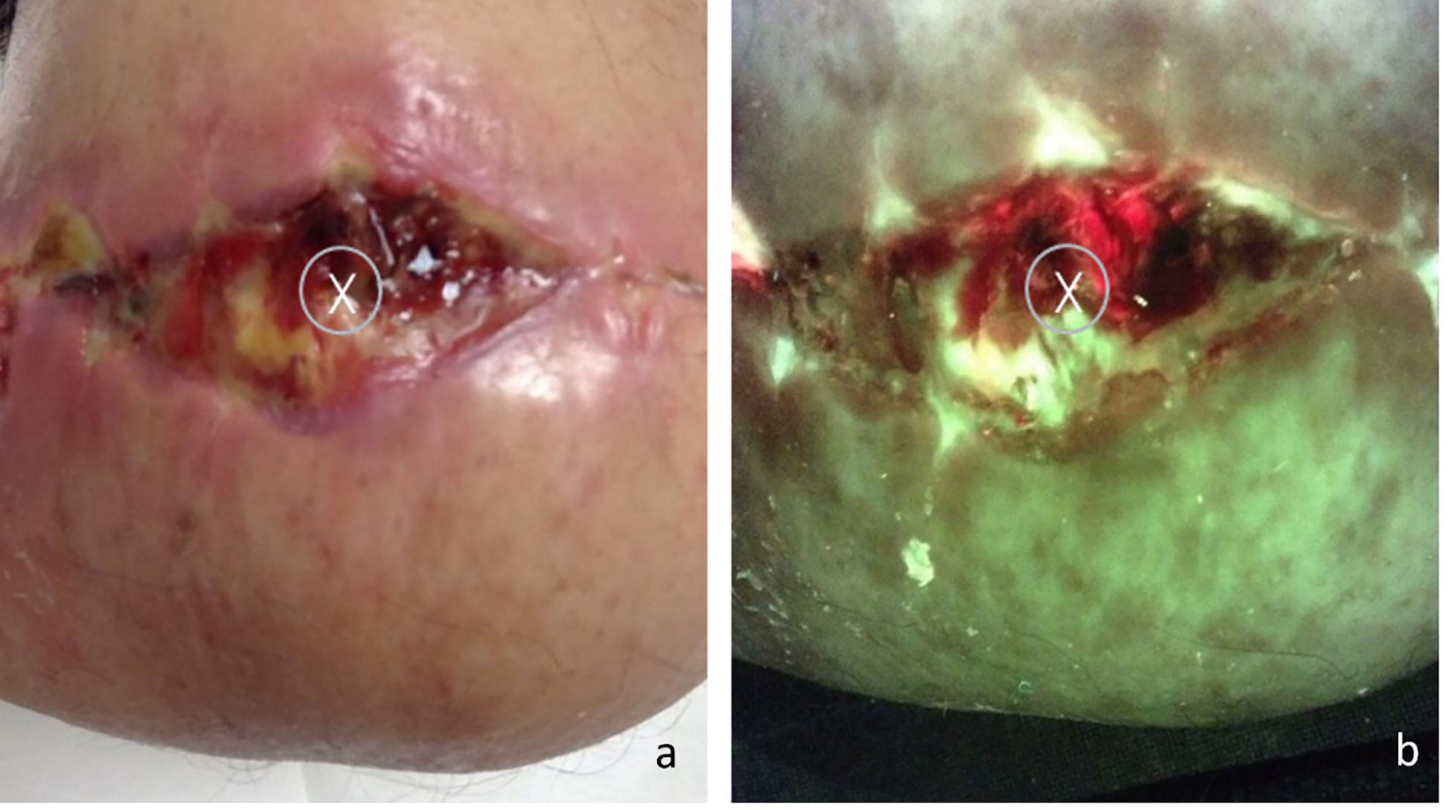
**(A)** Standard image of a stump wound, “X” marks the sample site as indicated by standard of care (center of the wound); **(B)** Fluorescence image of the same wound, center of the wound displays a positive red fluorescence image, indicative of gram -/+ presence at loads >10^4^ CFU/g. The coincidental overlap between SoC and area positive for FL accounted for 30% of the single-biopsy subjects.

**Figure 3:**
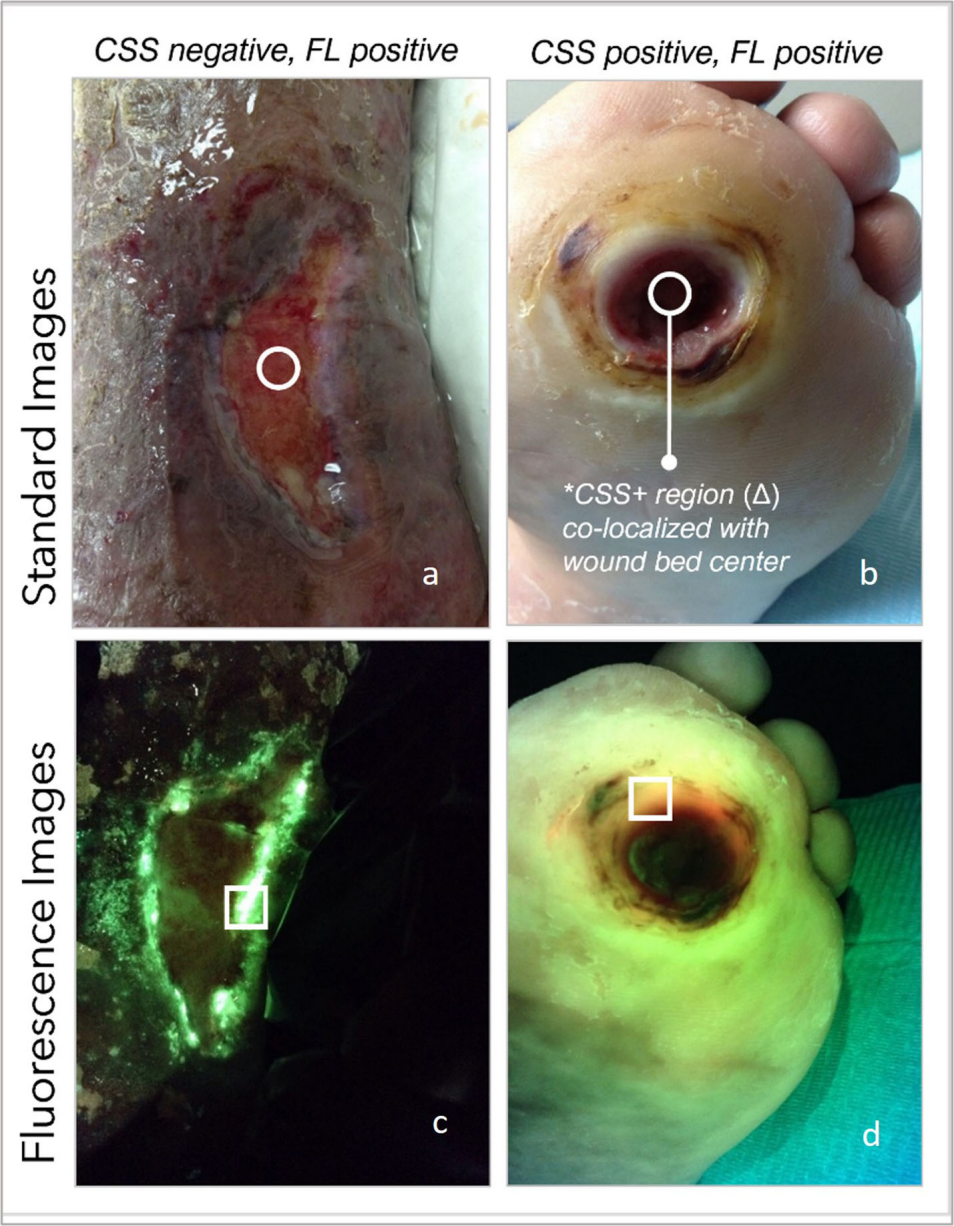
**(A)** Standard image of a wound deemed clinically unremarkable by the examining clinician, circle denotes location of SoC biopsy. **(B)** same wound under FL-imaging denotes areas positive for pseudomonas on edges of wound, far from SoC; **(C)** clinically remarkable plantar ulcer as per clinician’s assessment, on standard image; **(D)** same plantar wound denotes red FL-positive areas toward the edge of the wound, away from SoC location.

Analysis of the single-biopsy cohort (n=272) showed that 62 out of the 272 single-biopsies overlapped with areas of positive fluorescence signal. This means that SoC yielded samples with objective evidence of bacterial loads >10^4^ CFU/g in 29% of those wounds. **Figure 2** provides an example of one of the cases where this happened. On the opposite end of the spectrum, in the remaining 71% of wounds, FL-imaging often demonstrated bacterial presence far from the SoC site and frequently near the edge of the wound, as shown in **Figure 3**.

Next, **Table 2** depicts a comparison between the subset of the FLAAG trial that underwent a single-biopsy and the double-biopsy subset included in this *post hoc* analysis. As observed in this table, the wounds that warranted a second sample site were significantly wider (*p=*0.0083), longer (*p*<0.001), deeper (*p=*0.02) and had an overall near significant larger area (*p=*0.09) when compared to the population that underwent only one biopsy.

**Table 2:** Comparison of wound characteristics between single and double-biopsy cohorts.

Wound characteristics	Double-biopsy cohort	Single-biopsy cohort	Percentage change*	*p* value
**Wound Length (cm)**	4.01 SD(2.99) [0.50 - 16.10]	2.76 SD(2.88) [0.2, - 23.4]	45.30%	*P*<0.001
**Wound Width (cm)**	2.22 SD(2.50) [0.4 - 19.20]	2.03 SD(2.28) [0.2 - 23.5]	9.35%	*p*=0.0083
**Wound Depth (cm)**	0.42 SD(0.57) [0.00 - 3.00]	0.287 SD(0.42) [0.00,3.2]	50%	*p*=0.02
**Wound Area (cm^2^)**	14.96 SD(23.21) [0.50 - 144.00]	9.78 SD(24.09) [0.08 - 270.25]	52.90%	*p*=0.09

* The percentage change between the mean measurement in the single-biopsy and the double-biopsy cohort respectively.

### 3.2 Pathogens detected

The sensitivity to detect and localize high bacterial loads – thereby pointing to regions likely to yield a representative sample containing at least some pathogens of interest present in that wound – was high at 87.2% (95% CI: 77.7%, 93.7%) for the SoC-guided biopsy, while the sensitivity of the FL-guided biopsy was higher at 98.7% (95% CI: 58.7%, 99.8%) (*p=*0.0059).

**Figure 4:**
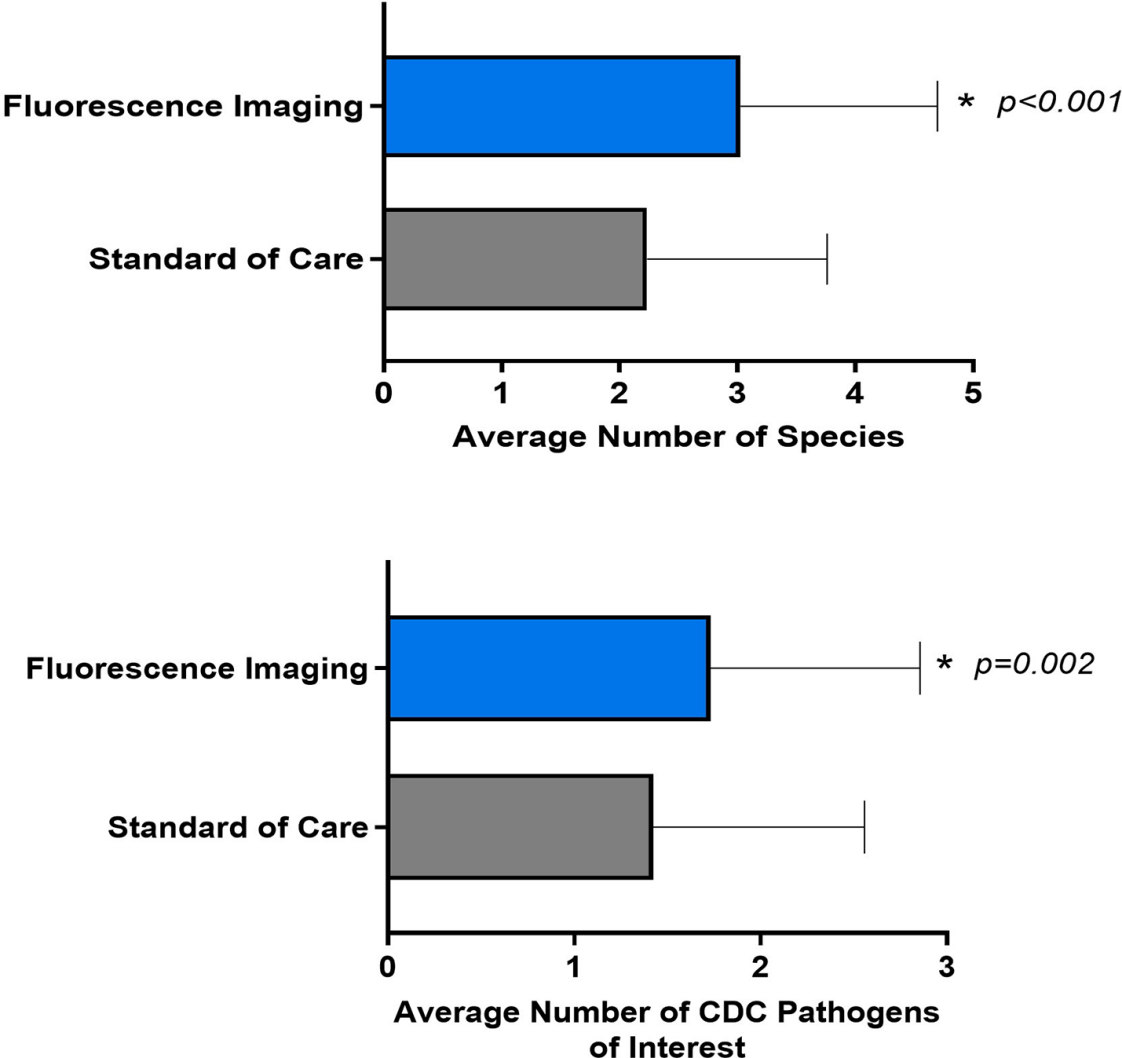
Average number of species and CDC pathogens of interest detected by fluorescence imaging compared to standard of care. Error bars indicate standard deviation. P values are based on paired t-tests.

Both biopsy methods yielded a significant number of aerobe and anaerobe, Gram positive and Gram-negative bacteria (**Supplementary Table 1**). Analysis focusing on the specific microbiological findings of the samples demonstrated that the average number of pathogens detected through the FL-informed biopsies were higher than those guided by SoC, as depicted in **Figure 4**. The average number of species detected through SoC was 2.2 (SD 1.53, median 2, range: 0-6] and for biopsies taken at a FL-positive site (FL-informed), this increased to 3.03 (SD 1.67, median 3, range: 0 -8] (*p<*0.001).

Furthermore, while both sample sites produced generally similar results in terms of the typical species of pathogens detected (See **Table 3**), the number of CDC’s pathogens of concern, meaning those considered high-risk threats for antibiotic resistance (CDC, 2019a), detected through SoC was inferior when compared to those samples obtained from a FL positive site.

**Table 3:** Top 10 species identified in each sampling location.

Most common species from SoC-guided biopsy at wound bed center		Most common species at FL-guided biopsy location	
Name	Count	Name	Count
Staphylococcus aureus**	38	Staphylococcus aureus**	36
Enterococcus faecalis**	32	*Enterococcus faecalis*	32
Pseudomonas aeruginosa**	16	Pseudomonas aeruginosa**	16
*Streptococcus agalactiae*	11	*Streptococcus agalactiae*	9
*Enterobacter cloacae* complex*/**	9	Escherichia coli*/**	6
*Proteus mirabilis*	8	*Proteus mirabilis*	6
Escherichia coli*/**	5	*Enterobacter cloacae* complex*/**	5
*Morganella morganii*	5	*Morganella morganii*	5
*Klebsiella pneumoniae*	3	*Proteus hauseri*	2
*Serratia marcescens*	3	*Providencia rettgeri*	2

CDC’s list of antibiotic resistance threats, aka “pathogens of concern” (CDC, 2019a): *Urgent threat (highest level of concern), **Serious threat.

**Figure 5:**
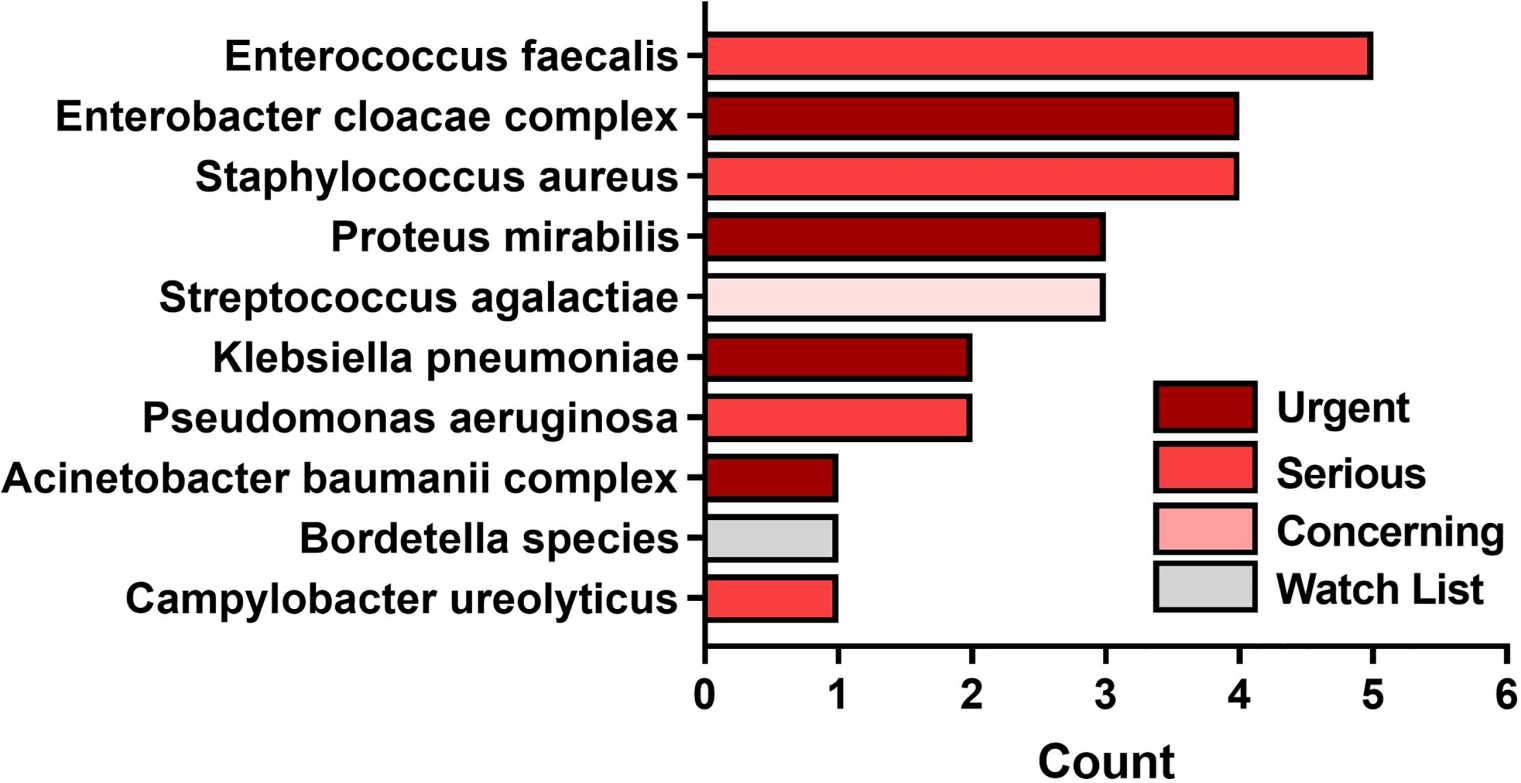
Most common pathogens detected by fluorescence guided biopsy that were missed during standard of care sampling at the center of the wound. Bars denote the number of FL informed biopsies where each pathogen was detected. Bar colors represent the 1 of 4 categories of CDC pathogens of concern per threat level.

The average number of SoC-detected species considered pathogens of concern or AR threats by the CDC was 1.4 (SD of 1.13; median of 1), while biopsies taken at a FL-positive site produced a significant increase to an average of 1.7 (SD of 1.12; median of 2). (*p=*0.002). **Figure 5** demonstrates the number of times that CDC pathogens of concern were missed by SoC-guided biopsies but were found through FL-informed sampling. *Enterococcus faecalis*, *Enterobacter cloacae*, and *Staphylococcus aureus* were the most frequently missed pathogens in SoC biopsies that were detected in biopsies from FL-positive regions.

## 4 Discussion

Heavy wound bacterial burden and biofilm presence are frequent in chronic, hard-to-heal wounds (Gjødsbøl K et al., 2006; Bjarnsholt et al., 2008; Dowd et al., 2008; Dowd et al., 2011; Lantis et al., 2013; Rahim et al., 2017). These bacterial communities are often polymicrobial and work together as a consortia further strengthening their resilience (Hurlow and Bowler, 2022). The presence of infection and/or critical levels of bacterial burden is widely recognized as a perpetuator of inflammation and as a healing deterrent (Leaper et al., 2012; Rahim et al., 2017). Proactive, local, and physically aggressive measures are considered most effective for the treatment of this interfering bacterial load, often found in biofilm form (Bowler et al., 2001; Bowler, 2002; Leaper et al., 2012; Cole and Coe, 2020; International Wound Infection Institute (IWII), 2022). Clinical guidelines support this notion, where local measures are the proposed first line of treatment (Apelqvist et al., 2000; Jones and Harding, 2015; International Wound Infection Institute (IWII), 2016; International Wound Infection Institute (IWII), 2022). Accurately mapping bacterial communities in order to effectively target them is therefore a critical piece of information for hygiene-based strategies (Cole and Coe, 2020; Moelleken et al., 2020). The advent of FL-imaging supports bacterial and biofilm localization and removal through frequent and targeted treatment, which should be the hallmark of skin and soft tissue infections (SSTI) and overall wound management (Wilcox et al., 2013; Lebrun E, 2013). There are several ways in which FL-imaging’s mapping of bacteria is relevant. It enables conservative debridement with minimal disturbance of healthy or newly re-epithelialized tissue by directing the procedure to the areas where it is needed. It also allows for the efficacy of a wound hygiene session to be assessed, as even extensive and prolonged sharp debridement can leave bacteria behind (Raizman et al., 2019; Cole and Coe, 2020; Moelleken et al., 2020). Additionally, it alerts towards the invasive extension of critical bacterial presence beyond the wound bed (i.e. wound-related cellulitis), which can be clinically unremarkable but result in serious complications if left untreated (Andersen et al., 2022). When skin grafting is needed, precisely identifying and eliminating bacterial hotspots can help prevent graft failure due to bacterial colonization at the grafting site (Unal et al., 2005; Donegan et al., 2014; Blumenthal and Jeffery, 2018; Blumenthal and Jeffery, 2018; Ai-Jalodi O et al., 2021); choosing the healthiest (less colonized) graft site greatly increases the take-rate (Unal et al., 2005).

Chronic wounds have unique, polymicrobial and patient-specific microbiological profiles that cannot be inferred merely from patient’s demographics or wound type. A wound’s microbiome, bacterial virulence, and resistance genes can vary significantly between patients (depending on geographical location and comorbidities, for example) and even between wounds on the same patient (Wolcott et al., 2016; Dekker et al., 2021). Further, the traditional concept of *a single, main offending pathogen* may not apply in the context of a chronic wound (Rhoads et al., 2012; Rhoads et al., 2012). This suggests that when an accurate microbiological diagnosis is required the sample from which it comes from must provide an adequate representation of the patient’s specific wound’s microbiome. Selecting the most representative sample site can prove challenging if the location of the bacterial loads is unclear. As evidenced by the findings of the present study, clinical uncertainty in sample site selection grows as the wounds become larger, and more possible sites of interest coexist. In larger wounds, where the likelihood of missing bacterial hotspots was greater, FL-informed biopsies had increased sensitivity. Not only were more areas of high bacterial loads found, but a significantly higher number of pathogen species per sample were detected, including those considered antibiotic resistance threats.

Traditionally, microbiological analysis has a number of inherent limitations often linked to the absence of an objective method that flags the location of pathogenic bacterial presence in real-time during collection. Sampling techniques are highly variable between operators and are based on clinical assessment, which is a somewhat subjective measure (Haalboom et al., 2019). Current guidelines propose CSS as the guide towards the appropriate sample site. But there seems to be a disconnect between the well-recognized phenomenon of attenuated CSS in hard-to-heal wounds and the recommendations to proactively diagnose and treat high bacterial loads locally based on them. Additionally, microbiological reports are delayed, sometimes by several days. When the report doesn’t yield complete information, and in the absence of adequate and timely results, the window to effectively treat could close entirely. The indications for sampling can also be susceptible to misinterpretation as chronic wound assessment and treatment has not been fully standardized. In fact, sampling can also be overutilized unnecessarily, particularly the swabbing of non-healing wounds. This leads to resource misuse, such as over-prescription of antibiotics (Gürgen, 2014).

When sampling is indicated and appropriate, FL-imaging facilitates a more comprehensive and accurate localization of microbial “hotspots” in real time, during sample collection. A study by Ottolino-Perry et al. demonstrated that FL-imaging guidance enhanced the diagnostic accuracy of wound swabs in a DFU cohort when compared to the Levine technique (Ottolino-Perry et al., 2017). Other authors have demonstrated its usefulness in enhancing the sensitivity, specificity, PPV, and NPV of the results obtained through swabs of burn wounds (Blumenthal and Jeffery, 2018; Farhan and Jeffery, 2019). In the present study, the enhancement of the quality of the samples was represented by a statistically significant increase in the number of pathogens detected through FL-guided sampling, including pathogens considered antibiotic resistance threats by the CDC (Center for Disease Control in the US) and the WHO (World Health Organization) (WHO, 2017; CDC, 2019a). Identifying specific pathogens is highly relevant in many cases in order to alert to the need for urgent, targeted measures. For instance, certain bacterial species have a higher propensity to form biofilm, leading to higher complication rates and poorer outcomes. *Pseudomonas aeruginosa*, which uniquely emits a cyan color on fluorescence images, has been linked to delayed healing, a skin graft failure rate of over 90%, serious infection, and increased mortality in venous leg ulcers and burns (Tredget et al., 1992; Unal et al., 2005; Gjødsbøl K et al., 2006). Antibiotic-resistant strains of *P. aeruginosa* have caused outbreaks across entire burn units (Aguilera-Saez et al., 2019).

Enhancing bacterial diagnostic techniques to augment clinical expertise and experience may hold a key to greater therapeutic success (Xu and Hsia, 2018). With this in mind, *Diagnostic Stewardship* has been proposed as an essential partner to adequate resource allocation, including antibiotic stewardship (Fatemi and Bergl, 2022; Patel and Fang, 2018; Sullivan, 2021) and is defined by the WHO’s Global Antimicrobial Resistance Surveillance System as *“coordinated guidance and interventions to improve appropriate use of microbiological diagnostics to guide therapeutic decisions. It should promote appropriate, timely diagnostic testing, including specimen collection, and pathogen identification and accurate, timely reporting of results to guide patient treatment”* (WHO, 2016). In a broad sense, diagnostic stewardship entails understanding which diagnostic tools benefit a particular clinical query in a specific individual, when to best utilize these tools, and how to interpret their results appropriately for optimal clinical conduct. In wound care, the search for a reliable biomarker that allows for early intervention without depending on an overt expression of inflammation or infection is underway. Several biomarkers for early detection of high bacterial presence have been proposed, including protease-based detection (Heinzle et al., 2013; Serena TE and Brosnan, 2022), a Strep A rapid swab test (Close et al., 2022), rapid enzyme analysis/activity detection (Hajnsek et al., 2013; Blokhuis-Arkes MH et al., 2015), a capsulized vesicle activated by bacterial toxins (Young AE et al., 2020), pH and uric acid monitoring bandages (Pal et al., 2018), and volatile organic compound detection (Ashrafi M et al., 2017). Though promising, some are still experimental, are technically challenging, lack portability or accessibility, or require invasive measures – and few provide bedside results. In contrast, most of these shortcomings are overcome by FL-imaging.

The goal of diagnostic stewardship is to improve patient outcomes. In the case of FL-imaging, this has been demonstrated through proven improvement in healing rates. A recent RCT reported a 2-fold increase in healing rates of DFUs intervened with a FL-imaging device throughout different stages of care. This included the initial assessment and subsequent evaluations (Rahma et al., 2022). This RCT’s results were in line with a previous study by Price et al. where healing rates were improved by over 20% after incorporating this technology into their DFU outpatient assessments (Price, 2020). The latter study also quantified the change in resources, including systemic antibiotic use before and after the advent of FL-imaging. Systemic antibiotic prescription and in general, resource utilization was reduced overall. These findings are particularly interesting, as they suggest that optimization of antibiotic use is related to better outcomes, in line with diagnostic stewardship principles. As mentioned previously, chronic wounds have established and resilient bacterial communities often in biofilm form, that are often unaffected by systemic antibiotics unless physically disturbed (Malic et al., 2011; Agostinho et al., 2011; Percival et al., 2011; Dalton et al., 2011). In that sense, local therapies focused on local removal of bacteria should be favored (Bowler et al., 2020; Hurlow and Bowler, 2022). On certain occasions where a microbiological diagnosis is needed (an overlaying acute infection on a chronic wound (Hurlow and Bowler, 2022), a wound-related complication like cellulitis (Andersen et al., 2022), or the wound bed preparation for a CTP or skin graft placement (Aung, 2019), etc.) it is important to ascertain the microbial composition of the affecting pathogens. And, when targeted antibiotic prescription is needed, it is imperative to utilize them in the most rational manner possible.

Evidence shows that the correlation between CSS and antibiotic administration is poor, mainly due to the lack of a straightforward, universal method of assessing a wound appropriately, but also because there may be a misunderstanding in the usefulness of systemic antibiotics in chronic wounds. Systemic antibiotics, and consequently tissue sampling and microbiological analysis, are most helpful in the context of an acute infection, which can stem from a chronic wound, but this is not universally understood. Antibiotic use rates in SSTIs are staggering and warrant close monitoring and use reduction strategies. More than 2 million U.S. patients per year are affected by SSTIs (Healthcare JCCfT, [[NoYear]]) and nearly half of all uncomplicated ambulatory care skin infections receive avoidable antibiotics (Hurley, 2013). In burn wounds, empirical and pre-surgical prophylactic antibiotic therapy is ubiquitous despite a lack of evidence (Avni T et al., 2010; Hurley, 2013). In chronic wounds, systemic antibiotic use is both extremely common and haphazard (Serena et al., 2022). Chronic wounds account for 16.5% of all antibiotic prescriptions in the UK and multiple patient series globally report rates of antibiotic prescription, at some point during their outpatient wound care, between 53%-71% (Tammelin et al., 1998; Howell-Jones et al., 2006; Öien and Forssell., 2013; Gürgen, 2014; Price, 2020). Strategies to promote rational prescription and to combat antibiotic resistance include enhancing awareness through national guidelines, CDC and WHO educational initiatives, antimicrobial use registries, and antimicrobial stewardship programs (CDC, 2019a). Despite these efforts, adequate and rational use of antimicrobials has continued to be an elusive goal (CDC, 2019b), particularly in the field of wound care. Promisingly, a 2020 study looking at the impact of fluorescence imaging on foot ulcer wound management reported a 33% decrease in patients prescribed systemic antibiotics for their wound (from 67% to 45%) and a 49% decrease in the percent of patients prescribed topical antimicrobials (from 85% to 44%) (Price, 2020). This was attributed to earlier bacterial detection – a diagnostic improvement – and more thorough bacterial removal through targeted debridement. Thus, FL-imaging can have a more global role; it can aid in the evaluation of the state of a wound and help determine the need and location of debridement, tissue sampling, and all procedures where bacterial mapping is of importance.

## 5 Conclusions

The indications and techniques to perform wound tissue sampling for microbiological analysis are susceptible to improvement and refinement. Limiting its implementation to when it’s truly indicated (e.g., grafting preparation, secondary acute infections, etc.) and objectively selecting the ideal sample location are areas worth improving upon. This motivation to better sampling techniques has been prompted by increasing evidence of the variability in tissue sampling methods in chronic, hard-to-heal wounds which result in unreliable microbiological and sensitivity reports that evolve into selection of inappropriate antimicrobial therapies (Gürgen, 2014) or costly losses of CTP and skin grafts (Unal et al., 2005). In addition, there is mounting evidence against the reliability of CSS-based approaches for early and covert bacterial presence detection (Church et al., 2006; Gardner et al., 2009; Le L et al., 2021; Rodríguez-Rodríguez et al., 2021). The current analysis objectively demonstrates that the addition of FL-imaging to complicated chronic wound evaluation aids in the identification of ideal tissue sample sites, resulting in more precise and complete wound microbial profile. This effect was particularly pronounced for larger wounds where there is likely more clinical uncertainty. Superior bacterial detection is in alignment with diagnostic and antibiotic stewardship goals for refinement of microbiological detection and therapeutic deployment, in wound care and beyond. A thorough evaluation of the clinical status of the wound has a great potential to decrease unnecessary antibiotic prescribing by promoting and enabling local measures of wound hygiene including debridement and topical antimicrobials; but also, by providing appropriate tissue samples that yield reliable microbiological results.

## 6 Limitations

This sample size of this subset did not allow for sub-group analysis (e.g., per type of wound). Additional limitations of the present *post hoc* analysis are derived from the original FLAAG clinical trial’s design which was to evaluate trial primary outcomes of imaging device diagnostic accuracy, and not to compare microbiological results between biopsy locations. However, we leveraged the occurrence of a 78 wound cohort that underwent 2 biopsies to analyze whether there were microbiological differences. The standardized sample acquisition protocol (biopsies, not swabs) was designed to serve the purposes of the original trial. Per trial design, samples could not be obtained after debridement as guidelines advise, as not all wounds were appropriate for debridement. The present study would be complemented by a prospective observational study adequately powered for subgroup analysis of sampling method and type of wound, where the sampling method is based on clinical necessity rather than a study protocol.

## Data availability statement

The data analyzed in this study is subject to the following licenses/restrictions: Contains patient information that can lead to identification. Raw data can be made available upon request. Requests to access these datasets should be directed to serena@serenagroups.com.

## Author contributions

Conceptualization, TS and PB; Methodology, TS; Writing—original draft preparation, all authors; writing—review and editing, All Authors. All authors have read and agreed to the published version of the manuscript.
