# Supplementary material for: Use of fluorescence imaging to optimize location of tissue sampling in hard-to-heal wounds
Source: Front Cell Infect Microbiol. 2023 Jan 12;12:1070311. doi: 10.3389/fcimb.2022.1070311 (PMC9878329; doi:10.3389/fcimb.2022.1070311)
Supplement: Supplementary file 1 [file Table_1.docx]

| Supplementary Table 1. List of *all* pathogens found during the collection of the 2-sample biopsies | | | | | | | |
| --- | --- | --- | --- | --- | --- | --- | --- |
| Pathogen | **No. of biopsies in which it was found** | | | **% of the total number of biopsies performed** |  |  |  |
| *Staphylococcus aureus* | | 75 | 97% | | aerobic | * | gram- |
| *Enterococcus faecalis* | | 62 | 81% | | aerobic | * | gram- |
| *Corynebacterium* species | | 47 | 61% | | aerobic | * | gram- |
| *Pseudomonas aeruginosa* | | 30 | 39% | | aerobic |  | gram- |
| *Streptococcus agalactiae* | | 20 | 26% | | aerobic | * | gram- |
| *Finegoldia magna* | | 20 | 26% | | anaerobic |  | gram+ |
| *Enterobacter cloacae* complex | | 14 | 18% | | aerobic | * | gram- |
| *Proteus mirabilis* | | 14 | 18% | | aerobic | * | gram+ |
| *Staphylococcus epidermidis* | | 13 | 17% | | aerobic | * | gram+ |
| *Escherichia coli* | | 11 | 14% | | aerobic | * | gram+ |
| *Morganella morganii* | | 10 | 13% | | aerobic | * | gram+ |
| *Corynebacterium striatum* | | 10 | 13% | | aerobic | * | gram- |
| *Bacteroides fragilis* | | 7 | 9% | | anaerobic |  | gram+ |
| *Klebsiella oxytoca* | | 7 | 9% | | aerobic | * | gram- |
| *Porphyromonas species* | | 5 | 6% | | anaerobic |  | gram+ |
| *Alcaligenes faecalis* | | 5 | 6% | | aerobic |  | gram+ |
| *Serratia marcescens* | | 5 | 6% | | aerobic | * | gram- |
| *Proteus hauseri* | | 4 | 5% | | aerobic | * | gram- |
| *Klebsiella pneumoniae* | | 4 | 5% | | aerobic | * | gram- |
| *Globicatella* species | | 4 | 5% | | aerobic | * | gram - |
| *Staphylococcus lugdunensis* | | 4 | 5% | | aerobic | * | gram+ |
| *Prevotella* species | | 4 | 5% | | anaerobic |  | gram- |
| *Peptoniphilus* species | | 3 | 4% | | anaerobic |  | gram- |
| *Enterococcus casseliflavus* | | 3 | 4% | | aerobic | * | gram+ |
| *Bacteroides thetaiotaomicron* | | 3 | 4% | | anaerobic |  | gram- |
| *Providencia rettgeri* | | 3 | 4% | | aerobic | * | gram- |
| *Streptococcus mitis* group | | 3 | 4% | | aerobic | * | gram+ |
| *Streptococcus* Group C | | 2 | 3% | | aerobic | * | gram+ |
| *Citrobacter freundii* complex | | 2 | 3% | | aerobic | * | gram - |
| *Staphylococcus hominis* | | 2 | 3% | | aerobic | * | gram- |
| *Citrobacter koseri* | | 2 | 3% | | aerobic | * | gram- |
| *Aerococcus* species | | 2 | 3% | | aerobic | * | gram+ |
| *Acinetobacter baumannii* | | 2 | 3% | | aerobic |  | gram+ |
| *Peptoniphilus asaccharolyticus* | | 2 | 3% | | anaerobic |  | gram- |
| *Anaerococcus* species | | 2 | 3% | | anaerobic |  | gram+ |
| *Prevotella bivia* | | 2 | 3% | | anaerobic |  | gram- |
| *Kocuria* species | | 2 | 3% | | aerobic | * | gram- |
| *Globicatella sanguinis* | | 2 | 3% | | aerobic | * | gram+ |
| *Corynebacterium jeikeium* | | 2 | 3% | | aerobic |  | gram+ |
| *Propionibacterium acnes* | | 2 | 3% | | anaerobic | ^ | gram+ |
| *Providencia stuartii* | | 2 | 3% | | aerobic | * | gram+ |
| *Arcanobacterium haemolyticum* | | 2 | 3% | | aerobic | * | gram+ |
| *Campylobacter ureolyticus* | | 1 | 1% | | anaerobic |  | gram+ |
| *Porphyromonas gingivalis* | | 1 | 1% | | anaerobic |  | gram- |
| *Trueperella bernardiae* | | 1 | 1% | | aerobic | * | gram+ |
| *Stenotrophomonas maltophilia* | | 1 | 1% | | aerobic |  | gram+ |
| *Streptococcus anginosus* | | 1 | 1% | | aerobic | * | gram+ |
| *Eubacterium aerofaciens* | | 1 | 1% | | anaerobic |  | gram+ |
| *Fusobacterium* species | | 1 | 1% | | anaerobic |  | gram+ |
| *Enterobacter aerogenes* | | 1 | 1% | | aerobic | * | gram+ |
| *Staphylococcus simulans* | | 1 | 1% | | aerobic | * | gram+ |
| *Actinotignum* species | | 1 | 1% | | anaerobic |  | gram+ |
| *Anaerococcus tetradius* | | 1 | 1% | | anaerobic |  | gram+ |
| *Streptococcus intermedius* | | 1 | 1% | | aerobic | * | gram+ |
| *Clostridium perfringens* | | 1 | 1% | | anaerobic |  | gram+ |
| *Lactobacillus* species | | 1 | 1% | | anaerobic | ^ | gram- |
| *Staphylococcus haemolyticus* | | 1 | 1% | | aerobic | * | gram+ |
| *Staphylococcus pettenkoferi* | | 1 | 1% | | aerobic) | * | gram- |
| *Gemella morbillorum* | | 1 | 1% | | anaerobic | ^ | gram+ |
| *Parvimonas micra* | | 1 | 1% | | anaerobic |  | gram+ |
| *Streptococcus gallolyticus* | | 1 | 1% | | aerobic | * | gram+ |
| *Leclercia adecarboxylata* | | 1 | 1% | | aerobic |  | gram- |
| *Arthrobacter* species | | 1 | 1% | | aerobic |  | gram+ |
| *Brevibacterium* species | | 1 | 1% | | aerobic |  | gram+ |
| *Acinetobacter baumanii complex* | | 1 | 1% | | aerobic |  | gram+ |
| *Streptococcus pyogenes* | | 1 | 1% | | aerobic | * | gram+ |
| *Bordetella* species | | 1 | 1% | | aerobic |  | gram- |

* Facultatively anaerobic

^ Oxygen tolerant
